# Can Esophageal Stiffness and Thickness Measurement by Transcervical Sonoelastography Predict Reflux Esophagitis?

**DOI:** 10.2174/0115734056462689260813104254

**Published:** 2026-08-24

**Authors:** Gökhan Aygün, Sercan Özkaçmaz, Fatma Durmaz, Mesut Özgökçe, Ilyas Dündar, Nurşen Toprak, Yaren Dirik, Mesut Aydın, Yeliz Dadalı, Harun Elçiçek

**Affiliations:** 1 Department of Radiology, University of Health Sciences Van Research and Training Hospital, Van, Türkiye; 2 Department of Radiology, Van Yuzuncu Yıl University School of Medicine, Van, Türkiye; 3 Department of Gastroenterology, Ministry of Health Siirt Training Hospital, Siirt Training Hospital, Siirt, Türkiye; 4 Department of Gastroenterology, Health Sciences University Izmir Tepecik Eğitin ve Araştırma Hastanesi, Izmir, Türkiye; 5 Department of Radiology, Kırşehir Ahi Evran University, Faculty of Medicine, Kırşehir, Türkiye

**Keywords:** Ultrasound, Shear-wave elastography, Thickness, Stiffness, Gastroesophageal reflux, Reflux esophagitis, Shear Wave Velocity (SWV)

## Abstract

**Objective:**

We aimed to investigate the efficacy of transcervical esophageal sonoelastography in discriminating between patients with and without Reflux Esophagitis (RE).

**Methods:**

Patients who underwent an esophagogastroduodenoscopy following a transcervical sonoelastographic examination in a tertiary center were included. The thickness and Shear Wave Velocity (SWV) of the submucosa-mucosa, muscularis mucosae, and total esophageal wall were measured by using sonoelastography. Patients were classified into RE-positive (grades A-D according to The Los Angeles Classification) and RE-negative groups based on endoscopic findings. The elastosonographic features of RE (-) and RE (+) patients were compared statistically

**Results:**

Among the 503 included patients, 129 (25.6%) were RE (+) [(80 grade A (62%), 28 grade B (21.7%), 12 grade C (9.3%), 9 grade D (7%)] and 374 were RE (-). A 31.7% positive correlation between age and Total Wall Thickness (TWT) and a 21% negative correlation between TWT and submucosa-mucosa SWV were detected. The thickness of submucosa-mucosa, muscularis propria, and total wall were significantly higher in RE (+) patients compared to RE (-) patients (*p* < 0.001 for all). The SWVs of submucosa-mucosa, muscularis propria, and total wall were significantly lower in RE (+) patients compared to RE (-) patients (*p* < 0.001 for all). A cut-off value of 2.35 mm for TWT yielded 67% sensitivity and 57% specificity, while a cut-off of 2.08 m/s for total wall SWV yielded 79% sensitivity and 81% specificity for the diagnosis of RE.

**Discussion:**

The significant increase in cervical esophageal wall thickness in RE (+) is consistent with the literature. However, the markedly decreased stiffness is a novel finding demonstrated by this study.

**Conclusion:**

The evaluation of esophageal thickness and stiffness using sonoelastography may be added to routine neck ultrasound scans for the prediction of RE.

## 1 INTRODUCTION

Gastroesophageal Reflux Disease (GERD) is a growing health problem worldwide and is most common in Western and Northern countries [1, 2]. The esophageal complications of gastroesophageal reflux include Reflux Esophagitis (RE), bleeding, anemia, peptic ulcer, peptic strictures, dysphagia, Barrett’s esophagus, and gastroesophageal neoplasms. It can also lead to various extra esophageal conditions, such as dental erosion, laryngitis, cough, asthma, sinusitis, and even pulmonary fibrosis. Reflux esophagitis is one of the most common complications, characterized by injury to the esophageal mucosa secondary to the reflux of gastric fluids into the esophageal lumen [3].

The diagnosis of GERD is based on 24-hour esophageal pH monitoring, esophagogastroscopic examination results, and clinical signs and symptoms that are relieved by antireflux drugs [4]. Radiological modalities have limited value in the initial diagnosis. Fluoroscopic esophagography is a feasible and inexpensive method, but it does not correlate well with the frequency and severity of gastroesophageal reflux. A thickened distal esophageal wall (> 5 mm) presenting with a target sign composed of a contrast-enhancing mucosa and a hypoenhancing submucosa due to edema is the typical finding on contrast-enhanced CT or MRI [5]. There are a few transabdominal and endoscopic ultrasound studies focusing on the distal esophagus wall in the literature, and only one transcervical ultrasound study has examined the cervical esophageal wall for the prediction of RE [6, 7].

Given that it is an accessible method without contraindications, radiation exposure, or the need for contrast agents, we aimed to evaluate the diagnostic utility of transcervical sonoelastography by comparing the thickness and elasto values of the upper esophageal layers between RE (+) and RE (-) patients.

## 2 MATERIALS AND METHODS

This prospective study was approved by the Van Yuzuncu Yıl University, Türkiye Ethics Committee on May 18, 2022 (Approval number 08). Informed consent was obtained from all patients.

### 2.1 Patients

Patients aged ≥ 18 years who underwent a transcervical ultrasound for the evaluation of the cervical esophagus and neighboring structures prior to an esophagogastroduodenoscopy on the same day, between June 2022 and January 2023, at a tertiary university hospital were consecutively included in this prospective study.

Patients with anatomical (*e.g*., kyphoscoliosis), degenerative (*e.g*., intervertebral disc pathologies), rheumatological (*e.g*., ankylosing spondylitis, scleroderma, rheumatoid arthritis) or traumatic conditions (*e.g*., history of neck surgery or trauma) affecting neck posture were excluded. Additionally, patients with a history of radiation therapy to the chest, head, or neck region; marked thyroid or parathyroid lesions compressing the esophagus; previous or current history of esophageal or gastric neoplastic lesions/diverticula; clinical psychological conditions; or current infectious pharyngitis were not included in the study. Furthermore, patients with a history of using anti-ulcer medications, antireflux drugs, or other medications that could affect esophageal and gastric motility within one month prior to the examination day were excluded.

### 2.2 Gray-Scale Ultrasound and Elastographic Examination

After a 12-hour fast, sonographic examinations were performed by a single radiologist with 5 years of experience in neck ultrasound and elastography, using a Siemens ACUSON S2000™ (Siemens Healthcare, Erlangen, Germany) device. Acoustic Radiation Force Impulse (ARFI) elastography examinations were conducted using the Virtual Touch IQ method with a linear 9L4 probe while the patient was in a neutral supine position without any supporting material.

First, the entire neck and paraesophageal structures were screened on gray-scale ultrasound to ensure optimal localization and evaluate for exclusion criteria. Following optimal localization, the esophagus was visualized in the sagittal plane at the left lateral aspect, extending from the level of the thyroid cartilage to the manubrium sterni (Fig. **1**). Next, the thickness of the muscularis propria and the mucosa-submucosa was measured 1.5–2 cm caudal to the level of the thyroid isthmus (Fig. **2**). The average of the measurements for these layers was then calculated. The total wall thickness was measured from the inner edge of the mucosa to the outer edge of the muscularis propria. The means of all three measurements were recorded.

Elastographic measurements were performed at the same locations as the thickness measurements, maintaining the same patient position and utilizing the same probe. During the examination, patients were instructed to hold their breath and to refrain from coughing, swallowing, or moving. A Region of Interest (ROI) with an approximately 25–30 mm craniocaudal length, fully covering all layers of the anterior esophageal wall, was drawn manually (Fig. **3**).

An equal number of small square sampling boxes (1 x 1 mm) were placed in both the submucosa-mucosa and the muscularis propria. The boxes were placed in close proximity to one another within the most homogeneous areas (Fig. **4**). At least three (and up to five) closely spaced measurements were obtained for both the muscularis propria and the mucosa-submucosa. The mean SWVs for the submucosa-mucosa and muscularis propria were then calculated. The total wall SWV was calculated as the arithmetic mean of the submucosa-mucosa and muscularis propria SWVs.

All elastosonographic examinations were performed by a single operator with five years of experience in elastography and cervical ultrasonography.

### 2.3 Endoscopy and Los Angeles Classification

Endoscopic examinations were performed by two gastroenterologists, with four and eight years of experience respectively, who were blinded to the ultrasonographic measurements. Esophagogastroscopy was performed 15–120 minutes (mostly 30–45 minutes) following the ultrasonographic examination. During this interval, patients did not eat, drink, or take any oral medications.

Based on endoscopy, patients were classified as normal (without reflux esophagitis) or as having reflux esophagitis (grades A–D) according to the Los Angeles Classification [8].

Grade A: At least one mucosal break with a length of ≤ 5 mm that does not extend between two mucosal folds.

Grade B: At least one mucosal break with a length of > 5 mm that does not extend between the tops of two mucosal folds.

Grade C: At least one mucosal break that extends between two or more mucosal folds, but which involves < 75% of the esophageal circumference.

Grade D: At least one mucosal break that involves ≥ 75% of the esophageal circumference.

Patients without reflux esophagitis were classified into Group N; patients with reflux esophagitis were classified into Group RE, which was further subdivided into Group A (grade A), Group B (grade B), Group C (grade C), and Group D (grade D).

### 2.4 Statıstıcal Analysıs

Descriptive statistics for continuous variables were expressed as mean ± standard deviation, minimum, and maximum, while categorical variables were presented as Numbers (N) and percentages (%). Sex-disaggregated data are reported in accordance with the Sex and Gender Equity in Research (SAGER) guidelines [9]. Patients were classified as male or female based on their reported biological sex, and all relevant outcome measures (esophageal wall thickness, shear wave velocity, and prevalence of reflux esophagitis) were analyzed and reported separately by sex. For comparisons of group means regarding continuous variables, a one-way Analysis of Variance (ANOVA) was used. Duncan's multiple range test was used to evaluate the means of the different groups. Pearson correlation coefficients were calculated to analyze the correlations between these variables within each group. Additionally, a Receiver Operating Characteristic (ROC) analysis was performed to calculate the diagnostic efficiency of the variables in discriminating between groups. A *p*-value of < 0.05 was considered statistically significant. All statistical analyses were performed using SPSS software (IBM SPSS Statistics for Windows, Version 25.0. Armonk, NY: IBM Corp.).

## 3 RESULTS

A total of 503 patients with a mean age of 42.59 ± 14.14 years (range: 18–78 years) were included in this study. The male-to-female ratio was reported as 0.62 (192/311). The age difference between males (43.73 ± 13.23 years; range: 18–74) and females (41.88 ± 14.65 years; range: 18–78) was not statistically significant (*p* = 0.155) (Table **1**).

Among the 129 patients (25.6%) diagnosed with reflux esophagitis, 80 had grade A (62%), 28 had grade B (21.7%), 12 had grade C (9.3%), and 9 had grade D (7%) according to the Los Angeles Classification. The incidence of reflux esophagitis was 39.6% (76/192) in males and 17% (53/311) in females (Table **2**).

The total wall thickness in females (2.27 ± 0.47 mm; range: 1.0–3.9 mm) was significantly lower than in males (2.58 ± 0.55 mm; range: 1.4–4.3 mm) (*p* < 0.001).

The muscularis propria thickness in females (1.00 ± 0.25 mm; range: 0.3–1.8 mm) was significantly lower than in males (1.12 ± 0.30 mm; range: 0.5–2.2 mm) (*p* < 0.001).

The submucosa-mucosa thickness in females (1.28 ± 0.28 mm; range: 0.7–2.3 mm) was significantly lower than in males (1.46 ± 0.32 mm; range: 0.8–2.4 mm) (*p* < 0.001).

The muscularis propria SWV in females (2.28 ± 0.27 m/s; range: 1.59–3.3 m/s) was significantly higher than in males (2.06 ± 0.28 m/s; range: 1.24–2.99 m/s) (*p* < 0.001).

The submucosa-mucosa SWV in females (2.13 ± 0.26 m/s; range: 1.5–2.95 m/s) was significantly higher than in males (1.90 ± 0.27 m/s; range: 1.15–2.92 m/s) (*p* < 0.001).

The total wall SWV in females (2.20 ± 0.26 m/s; range: 1.57–3 m/s) was significantly higher than in males (1.98 ± 0.27 m/s; range: 1.19–2.96 m/s) (*p* < 0.001) (Table **3**).

The mean age of patients with RE (46.61 ± 13.97 years; range: 18–78) was significantly higher than that of patients without RE (41.2 ± 13.94 years; range: 18–77) (*p* < 0.001).

The total wall thickness of RE (+) patients (2.58 ± 0.61 mm; range: 1–4.3 mm) was significantly higher than that of RE (-) patients (2.32 ± 0.48 mm; range: 1.3–4.0 mm) (*p* < 0.001).

The muscularis propria thickness of RE (+) patients (1.13 ± 0.33 mm; range: 0.3–2.2 mm) was significantly higher than that of RE (-) patients (1.01 ± 0.25 mm; range: 0.4–1.9 mm) (*p* < 0.001).

The submucosa-mucosa thickness of RE (+) patients (1.46 ± 0.34 mm; range: 0.8–2.4 mm) was significantly higher than that of RE (-) patients (1.31 ± 0.29 mm; range: 0.7–2.3 mm) (*p* < 0.001).

The muscularis propria SWV of RE (+) patients (1.93 ± 0.21 m/s; range: 1.4–2.36 m/s) was significantly lower than that of RE (-) patients (2.29 ± 0.26 m/s; range: 1.24–3.3 m/s) (*p* < 0.001).

The submucosa-mucosa SWV of RE (+) patients (1.79 ± 0.20 m/s; range: 1.25–2.34 m/s) was significantly lower than that of RE (-) patients (2.13 ± 0.26 m/s; range: 1.15–2.95 m/s) (*p* < 0.001).

The total wall SWV of RE (+) patients (1.86 ± 0.20 m/s; range: 1.37–2.35 m/s) was significantly lower than that of RE (-) patients (2.21 ± 0.25 m/s; range: 1.19–3.0 m/s) (*p* < 0.001). (Table **4**).

There was a 31.7% positive correlation between age and total wall thickness and a 21% negative correlation between total wall thickness and submucosa-mucosa SWV (Table **5**).

The mean age of Group C was significantly higher than that of Group N (*p* < 0.001).

The total wall thickness in Group C was significantly higher than in Group D (*p* < 0.001).

The muscularis propria thickness in Group A was significantly higher than in Group D (*p* < 0.001).

The muscularis propria thickness in Group C was significantly higher than in Group D (*p* < 0.001).

The submucosa-mucosa thickness in Group C was significantly higher than in Group N (*p* < 0.001).

The muscularis propria SWV in Group A (1.96 ± 0.20 m/s; range: 1.46–2.36 m/s) was significantly lower than in Group N (2.29 ± 0.26 m/s; range: 1.24–3.3 m/s) (*p* < 0.001).

The muscularis propria SWV in Group B (1.87 ± 0.22 m/s; range: 1.4–2.29 m/s) was significantly lower than in Group N (2.29 ± 0.26 m/s; range: 1.24–3.3 m/s) (*p* < 0.001).

The muscularis propria SWV in Group C (1.87 ± 0.19 m/s; range: 1.55–2.21 m/s) was significantly lower than in Group N (2.29 ± 0.26 m/s; range: 1.24–3.3 m/s) (*p* < 0.001).

The muscularis propria SWV in Group D (1.96 ± 0.21 m/s; range: 1.63–2.36 m/s) was significantly lower than in Group N (2.29 ± 0.26 m/s; range: 1.24–3.3 m/s) (*p* < 0.001).

The submucosa-mucosa SWV in Group A (1.80 ± 0.20 m/s; range: 1.25–2.34 m/s) was significantly lower than in Group N (2.13 ± 0.26 m/s; range: 1.15–2.95 m/s) (*p* < 0.001).

The submucosa-mucosa SWV in Group B (1.77 ± 0.20 m/s; range: 1.4–2.17 m/s) was significantly lower than in Group N (2.13 ± 0.26 m/s; range: 1.15–2.95 m/s) (*p* < 0.001).

The submucosa-mucosa SWV in Group C (1.71 ± 0.17 m/s; range: 1.44–2.01 m/s) was significantly lower than in Group N (2.13 ± 0.26 m/s; range: 1.15–2.95 m/s) (*p* < 0.001).

The submucosa-mucosa SWV in Group D (1.84 ± 0.22 m/s; range: 1.5–2.18 m/s) was significantly lower than in Group N (2.13 ± 0.26 m/s; range: 1.15–2.95 m/s) (*p* < 0.001).

The total wall SWV in Group A (1.88 ± 0.19 m/s; range: 1.37–2.35 m/s) was significantly lower than in Group N (2.21 ± 0.25 m/s; range: 1.19–3.0 m/s) (*p* < 0.001).

The total wall SWV in Group B (1.82 ± 0.21 m/s; range: 1.4–2.23 m/s) was significantly lower than in Group N (2.21 ± 0.25 m/s; range: 1.19–3.0 m/s) (*p* < 0.001).

The total wall SWV in Group C (1.79 ± 0.17 m/s; range: 1.5–2.11 m/s) was significantly lower than in Group N (2.21 ± 0.25 m/s; range: 1.19–3.0 m/s) (*p* < 0.001).

The total wall SWV in Group D (1.90 ± 0.21 m/s; range: 1.57–2.27 m/s) was significantly lower than in Group N (2.21 ± 0.25 m/s; range: 1.19–3.0 m/s) (*p* < 0.001) (Figs **5**-**8**) (Table **6**).

All other differences between the groups were found to be non-significant (Table **6**).

In the ROC analysis for total wall thickness, a cut-off value of 2.35 mm yielded 67% sensitivity and 57% specificity for the diagnosis of RE (Fig. **9**).

In the ROC analysis for total wall SWV, a cut-off value of 2.02 m/s yielded 79% sensitivity and 81% specificity for the diagnosis of reflux esophagitis (Fig. **10**).

## 4 DISCUSSION

The incidence of RE has been reported as 15.5% in Northern Europe [10], whereas Loffeld *et al*. [11] reviewed 11,691 patients who underwent esophagogastroscopy over a 10-year period and found a frequency of 14% in the Netherlands. In Türkiye, Bor *et al*. [12] reported a reflux esophagitis frequency of 22.8%, while Yılmaz *et al*. [13] reported 12.8% among their patients. Yamagishi *et al*. [14] reported that in a health check-up program, 20% of their participants (n = 160,983) exhibited symptoms and signs associated with reflux esophagitis. In contrast to these studies, we detected RE in 25.6% of our patients. This relatively higher rate may be due to the fact that RE has recently become a more common medical problem owing to various factors, including changing eating habits and increased physiological stress in daily life. In the literature, the majority of patients with RE present with grade A or B disease [15]. Consistent with these findings, more than 80% of our patients with RE were classified as grade A or B.

It is well documented that esophageal motility decreases with age [16], making gastroesophageal reflux more common in elderly individuals [17]. Consistent with the literature, the mean age of our RE (+) patients was statistically significantly higher than that of the RE (-) patients. Furthermore, the oldest average patient ages in our study were in Group C (52.3 years) and Group D (47.0 years), suggesting that not only the frequency but also the severity of RE increases with age.

Doğan *et al*. reported an age-related increase in distal esophageal wall thickness (1.56 ± 0.32 mm in patients > 40 years old *versus* 1.29 ± 0.24 mm in patients < 40 years old) [18]. Consistent with these data, we observed a 32% positive correlation between age and total wall thickness, a 21% correlation between age and muscularis propria thickness, and a 36% correlation between age and submucosa-mucosa thickness.

Regarding the Male-to-Female (M/F) ratio in RE (+) patients, Chang *et al*. [19] reported a ratio of 5.6:1 (among 2,044 patients), Loffeld *et al*. [11] reported 1.6:1 (among 11,691 patients), and Lee *et al*. [20] reported 7:1 (among 7,015 patients). Additionally, a meta-analysis reviewing 28 studies concluded that males have a 1.57-times higher risk of erosive esophagitis than females [21]. Consistent with the literature, the M/F ratio in our study was 1.43 (76/53) among RE (+) patients compared to 0.45 (116/258) among RE (-) patients, and this difference was statistically significant. As recommended by SAGER guidelines, sex-based differences in esophageal wall thickness and elastographic values were systematically assessed; both parameters differed significantly between males and females independent of RE status, suggesting sex should be considered a relevant biological variable in future sonoelastographic studies of the esophagus.

In healthy subjects, Zhu *et al*. [22] reported a mean cervical esophageal anterior wall thickness of 2.3 ± 0.3 mm (range: 1.3–4.1 mm); Kaçar *et al*. [23] reported 2.29 ± 0.38 mm (range: 1.57–4.0 mm); and Mateesn *et al*. [24] reported 2.8 ± 0.4 mm. Similarly, we found a mean total wall thickness of 2.32 ± 0.48 mm (range: 1.3–4.0 mm) in our RE (-) patients. Caletti *et al*. [25] and Changchien *et al*. [26] both reported a higher total wall thickness in the distal esophagus of RE (+) patients compared to RE (-) patients. Mine *et al*. [27] also reported higher total wall, submucosa-mucosa, and muscularis propria thicknesses in RE (+) patients relative to controls. They further demonstrated that all of these values decreased following 12 months of antireflux treatment. However, Kaçar *et al*. [23] found a significant increase in the cervical esophageal luminal diameter in RE (+) patients compared to RE (-) patients, but no significant difference in esophageal wall thickness. Inconsistent with their findings, we observed a statistically significant increase in the total wall, submucosa-mucosa, and muscularis propria thicknesses in RE (+) patients compared to RE (-) patients. As a supporting finding, the greatest difference in thickness relative to the normal group was observed in Group C.

To the best of our knowledge, there is only one study in the literature that has evaluated elastography in the context of RE. Suhara *et al*. [28] investigated the predictive value of elastosonography at the gastroesophageal junction in reflux esophagitis using a transabdominal approach. They examined 41 RE (+) and 61 RE (-) patients using strain elastography and found a statistically significantly greater degree of stiffness in RE (-) patients compared to RE (+) patients. Similarly, we found statistically significantly lower elastography values (SWV) in RE (+) patients compared to RE (-) patients across the submucosa-mucosa, muscularis propria, and total wall of the cervical esophagus. The rate of SWV decrease in the submucosa-mucosa (15.9%) was mildly higher than that in the muscularis propria (15.6%). Although the rates of decrease were similar overall, we hypothesize that the more pronounced decrease in the submucosa-mucosa layer is due to its innermost location, making it more susceptible to direct exposure from luminal gastric contents during reflux. This finding is also consistent with the observation that the rate of thickness increase in the submucosa-mucosa (12%) was mildly higher than in the muscularis propria (11.9%).

Among RE (+) patients, the lowest SWVs for the total wall and muscularis propria were observed in Group C, and for the submucosa-mucosa in Group B, although the values for this layer were very similar between Groups B and C. This suggests that elastography values tend to be lower in more advanced grades, with the exception of Grade D. Generally, tissue stiffness tends to decrease during acute inflammation due to edema (without fibrotic changes) and increase in chronic phases due to fibrosis formation. Among RE (+) patients, the highest SWVs across the total wall, submucosa-mucosa, and muscularis propria were observed in Grade D. We believe that the higher elastography values in Grade D may be the result of such a chronic fibrotic process. Theoretically, in the initial stages, the disease is less severe and leads primarily to mild inflammation and edema, resulting in decreased elastography values. Conversely, in advanced phases, the reflux becomes more prominent, causing moderate-to-severe inflammation accompanied by fibrotic changes in the esophageal wall, which manifest as increased elastography values. However, the sample size in the Grade D group was relatively small (n = 9), which may contribute to these seemingly contradictory results.

Another finding of this study was a 31.7% positive correlation between age and total wall thickness, alongside a 21% negative correlation between total wall thickness and submucosa-mucosa SWV. The positive correlation between age and total wall thickness is likely due to the fact that the incidence of reflux esophagitis increases with age. The negative correlation between total wall thickness and submucosa-mucosa SWV may be explained by the fact that wall edema simultaneously increases thickness and reduces elastography values, whereas fibrotic changes decrease thickness and increase elastography values.

Through ROC analysis, we identified a cut-off value of 2.35 mm for total wall thickness (yielding 67% sensitivity and 57% specificity) and 2.08 m/s for total wall SWV (yielding 79% sensitivity and 81% specificity). Although there was a statistically significant difference in total wall thickness between patients with and without reflux esophagitis, the absolute difference was small (less than 1 mm: 2.58 mm vs. 2.32 mm). Furthermore, both the sensitivity and specificity were relatively low. For this reason, measuring cervical esophageal thickness to predict reflux esophagitis must be performed very carefully by a highly experienced observer using standardized device settings. Image zooming or magnification during the elastosonographic examination may be useful in ensuring accurate and sensitive measurements. We suggest considering the addition of thickness measurements (which adds an average of 1 minute) and elastography measurements (which adds an average of 3 minutes) to routine neck ultrasound scans in centers where RE is prevalent. This practice may help mitigate the chronic effects of reflux esophagitis, particularly in asymptomatic patients, and could potentially prevent unnecessary gastroscopic procedures.

The literature contains contradictory results regarding the application of elastography in the kidney and pancreas. Particularly in chronic conditions affecting the kidneys (such as vesicoureteral reflux and ureteropelvic junction obstruction) and the pancreas (such as cystic fibrosis), lower shear wave elastography values have been reported compared to controls. This association has been attributed to variabilities in blood flow and fibrotic changes [29]. Furthermore, data regarding the use of elastography on luminal organs remain very limited. We hypothesize that the elastographic features of the distal esophagus and cervical esophagus may differ. Frequent irritation of the distal esophagus by gastric contents may lead to chronic fibrotic changes, whereas the relatively weaker effect of reflux on the upper esophagus may cause only mild edema or increased vascularization.

In the literature, there are very few studies addressing the diagnostic efficiency of elastosonography in luminal gastrointestinal organs [29-33]. Data regarding the sonoelastographic features of the esophagus in RE are highly inadequate and generally limited to changes in the distal esophagus and gastroesophageal junction. However, transabdominal sonographic evaluation of the distal esophagus and gastroesophageal junction poses a significant challenge, as these structures are typically located more than 7–8 cm below the skin surface, a depth at which elastography often fails to accurately measure tissue stiffness. Moreover, the positions of these structures can be markedly affected by the diaphragm, neighboring intestinal loops, and even cardiac pulsations. Additionally, various artifacts and resolution issues frequently occur when evaluating these deeper structures. A very detailed patient preparation is required for this approach, as even a small, gas-filled bowel loop or minor peristaltism in the distal esophagus or stomach can complicate the examination. In contrast, the transcervical approach for evaluating esophageal stiffness offers several advantages. It is more feasible because the cervical portion is superficial, permitting excellent visualization and measurement using a high-frequency transducer. Furthermore, in the cervical region, there are fewer neighboring tissues that might shift the esophagus's position or cause significant artifacts during the examination. Measurement via the transcervical approach is also easier and more reliable, as the peristalsis of the upper esophagus is partially under voluntary control, leading to fewer unwanted movements during the scan. Importantly, this approach does not require strict fasting and provides excellent visualization of all esophageal wall layers.

In recent years, technological developments in the medical field have advanced rapidly and continue to evolve. One of the most revolutionary breakthroughs has been the introduction of the Internet of Things (IoT) concept into medical practice. For example, a recently proposed smart pH sensor system has demonstrated an accuracy of approximately 99.25% in diagnosing gastroesophageal reflux disease [34]. Additionally, 3D printing technology is providing better diagnostic access, facilitating surgical planning, and improving overall healthcare delivery.

The application of 3D printing in medicine-bridging imaging, education, and clinical practice-has shown promising results in the management of gastrointestinal and colorectal diseases [35]. We believe that the elastosonographic evaluation of esophageal thickness and stiffness, potentially augmented by smart systems and 3D printing technology, represents an interesting and promising area for future research in the detection of gastroesophageal reflux disease.

## 5 STUDY LIMITATIONS

The major limitation of this study is the relatively small sample size, particularly in the Grade D group, which may have contributed to the somewhat contradictory results observed in that cohort. Additionally, we could not correlate our ultrasound findings with histopathological results. As another limitation, we did not evaluate differences in esophageal luminal diameter or total esophageal diameter between RE (+) and RE (-) patients. We also did not correlate the SWV of the distal esophagus with that of the cervical esophagus in our patient population. Furthermore, the sampling boxes used to measure stiffness were 1 x 1 mm in size. Because the thickness of the muscularis propria and mucosa-submucosa was < 1 mm in a few patients, we had to carefully center the boxes onto the layers to ensure minimal signal overflow. Finally, in cases of less severe RE, the upper portion of the esophagus may be minimally or not at all affected, meaning that transcervical measurements might remain unaltered.

## CONCLUSION

This study demonstrated statistically significant increases in wall thickness and decreases in elastography values of the esophageal layers in patients with RE, using a highly feasible transcervical ultrasound method. These findings, particularly the correlation between elastography values and RE severity grades, must be confirmed by further studies utilizing larger sample sizes. Additionally, future research should compare baseline elastography values with post-treatment (anti-reflux therapy) measurements in patients with RE.

The thickening of the distal esophageal wall can often present a diagnostic challenge when attempting to discriminate between a malignant condition and a benign inflammatory process, such as reflux esophagitis, on CT scans. Future studies may also compare cervical esophageal wall elastography values in patients with malignant distal esophageal thickening to those with benign thickening.

## DECLARATIONS

In this preliminary study, we consecutively included a total of 503 patients, a sample size comparable to previous studies regarding the esophagus discussed herein. We also rigorously excluded patients with various conditions that could have negatively confounded the results.

## Figures and Tables

**Fig. (1):**
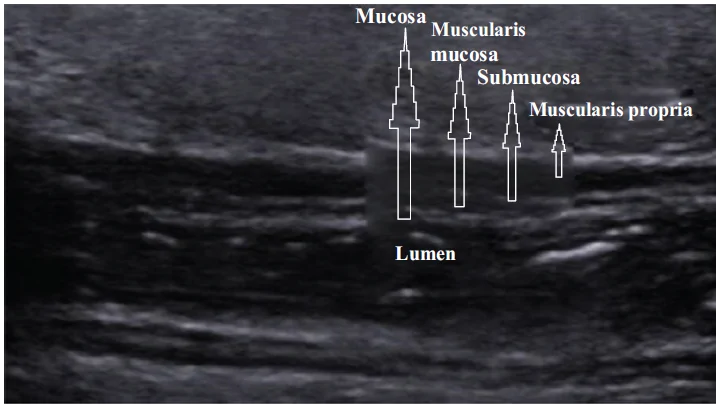
The demonstration of the layers of the esophagus on transcervical ultrasound: **1-Mucosa:**: The innermost layer, which is hyperechoic and relatively thicker; **2-Muscularis mucosa:**: Thin hypoechoic layer between mucosa and submucosa; **3-Submucosa:**: Thin hyperechoic layer between muscularis mucosa and muscularis propria; **4-Muscularis propria:**: Thick hypoechoic layer between submucosa and adventitia/serosa (very thin).

**Fig. (2):**
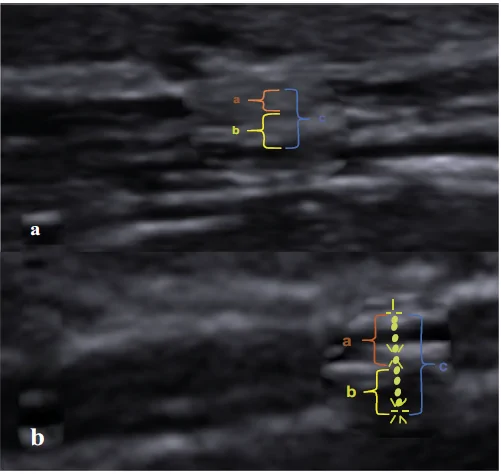
**(a, b)** Muscularis propria thickness measurement (orange-a); b) Submucosa-mucosa thickness measurement (yellow-b); c) Total wall thickness measurement (blue-c).

**Fig. (3):**
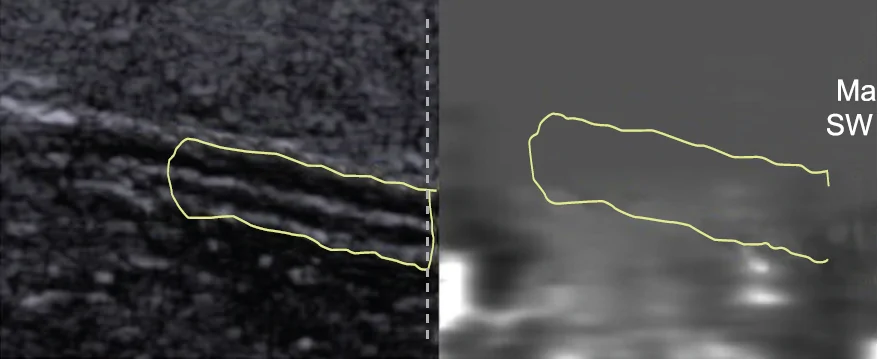
The ROI covering all layers of the anterior wall of the esophagus in gray mode (left) and the superimposed ROI on the image, which gives the quantitative elastography values (right).

**Fig. (4):**
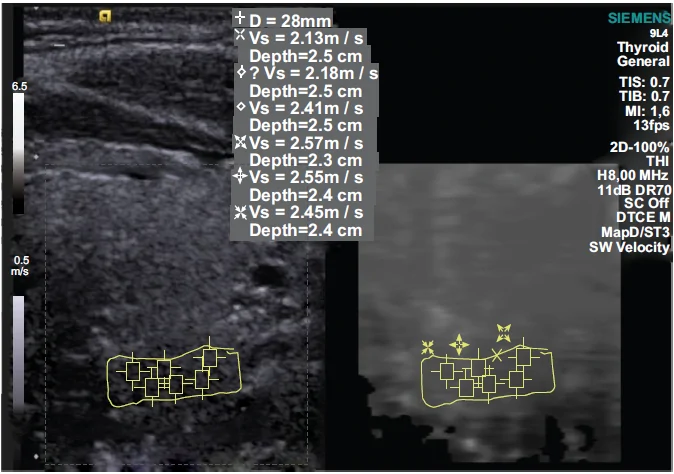
There are 3 boxes placed on the muscularis propria (2.45-2.55-2.57 m/sn) and 3 on submucosa-mucosa (2.13-2.18-2.41 m/sn).

**Fig. (5):**
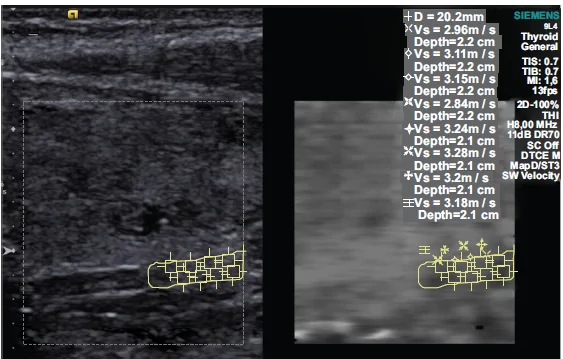
Elasto measurement of a 34-year-old female without RE.

**Fig. (6):**
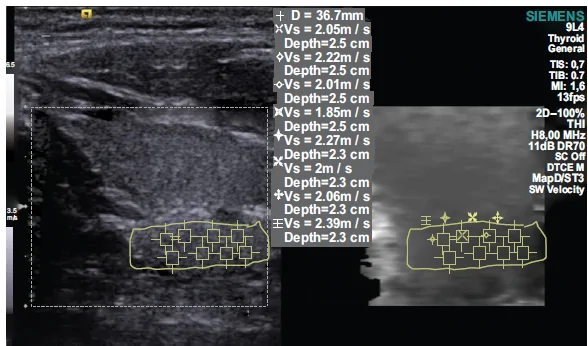
Elasto measurement of a 53-year-old male with Grade A RE.

**Fig. (7):**
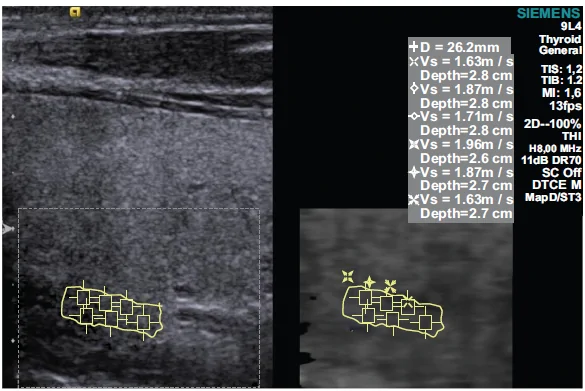
Elasto measurement of a 37-year-old male with Grade B RE.

**Fig. (8):**
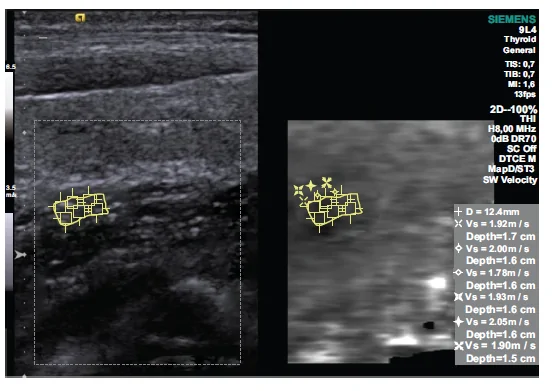
Elasto measurement of a 72-year-old female with Grade C RE.

**Fig. (9):**
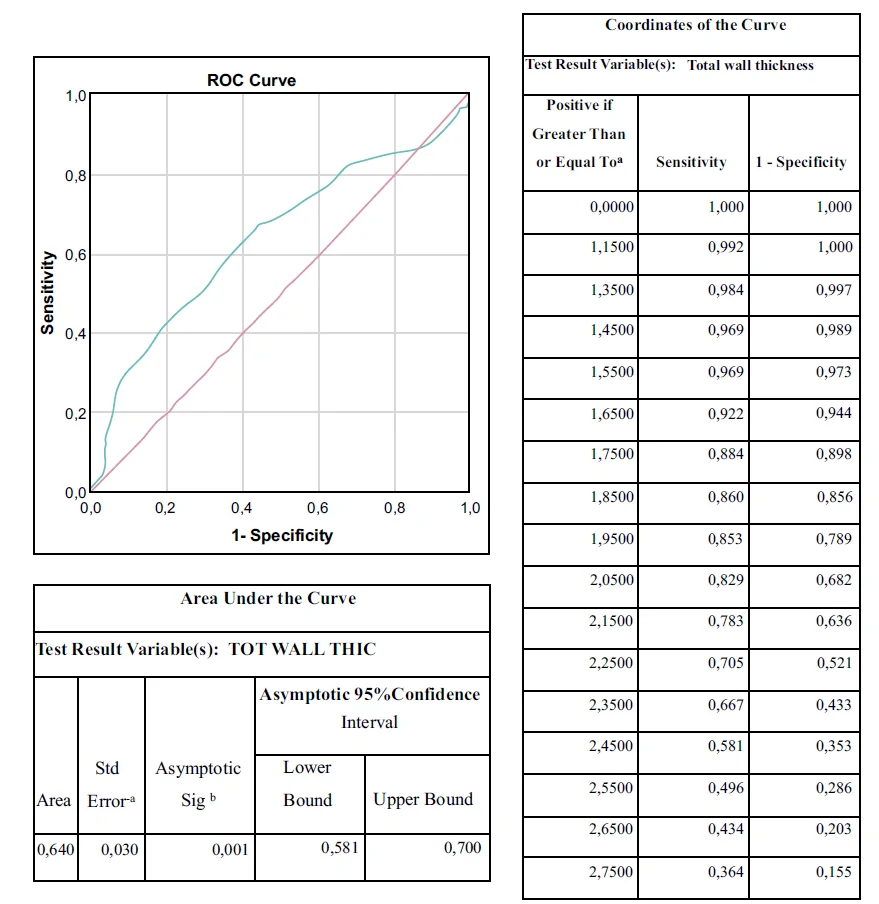
ROC analysis for the total wall thickness.

**Fig. (10):**
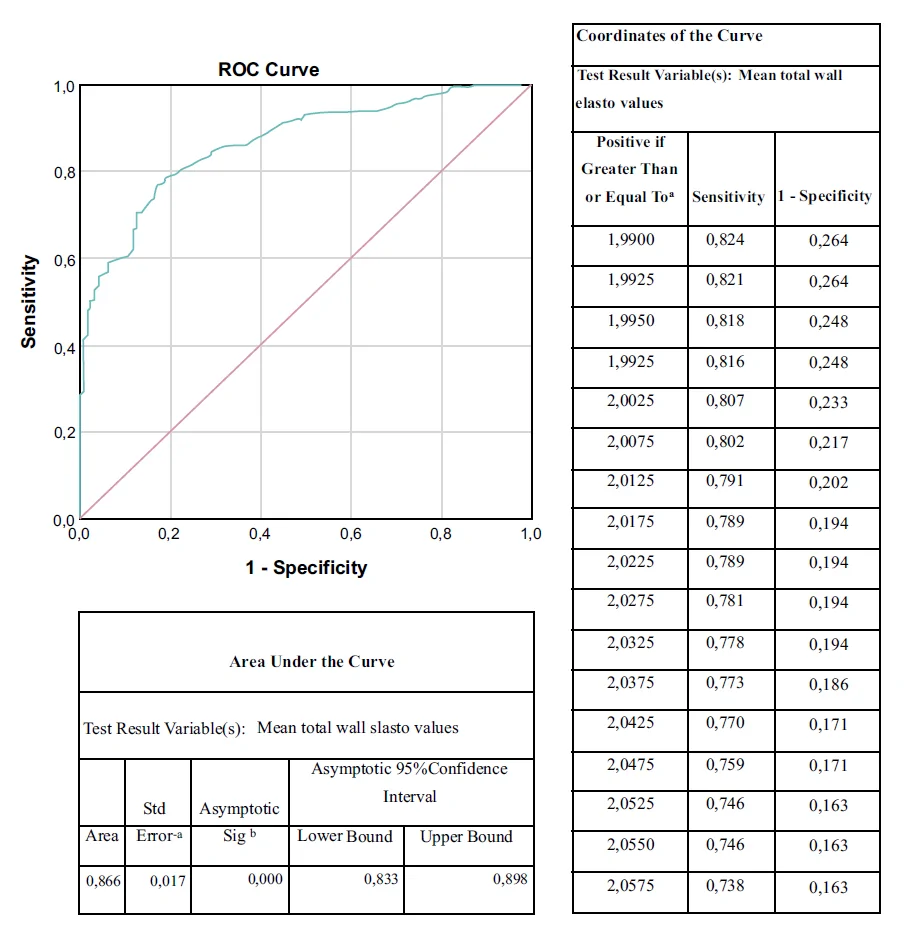
ROC analysis for the total wall SWV.

**Table 1:** Comparison of the RE (+) and RE(-) patients regarding sex.

-	-	**Sex**		**Total**
		**Male**	**Female**	
**Group N** **(Reflux Esophagitis (-))**	Number	116	258	374
	Intragroup	31.0%	69.0%	100.0%
	Intrasex	60.4%	83.0%	74.4%
	Total	23.1%	51.3%	74.4%
**Group RE** **(Reflux Esophagitis (+))**	Number	76	53	129
	Intragroup	58.9%	41.1%	100.0%
	Intrasex	39.6%	17.0%	25.6%
	Total	15.1%	10.5%	25.6%
**Total**	Number	192	311	503
	Intragroup	38.2%	61.8%	100.0%
	Intrasex	100.0%	100.0%	100.0%
	Total	38.2%	61.8%	100.0%
		**Chi-Square = 31.63 *p* < 0.001**		

**Table 2:** Comparison of the RE (-) and subgroups of RE (+) patients regarding **sex**.

-	-	**Sex**		**Total**
		**Males**	**Females**	
**Group N** **(RE (-))**	**Number**	116	258	374
	**% (subgroup)**	31.0	69.0	100.0
	**% (sex)**	60.4	83.0	74.4
	**Total**	23.1	51.3	74.4
**Group A** **(Grade A RE)**	**Number**	49	31	80
	**% (subgroup)**	61.3	38.8	100.0
	**% (sex)**	25.5	10.0	15.9
	**Total**	9.7	6.2	15.9
**Group B** **(Grade B RE)**	**Number**	15	13	28
	**% (subgroup)**	53.6	46.4	100.0
	**% (sex)**	7.8	4.2	5.6
	**Total**	3.0	2.6	5.6
**Group C** **(Grade C RE)**	**Number**	10	2	12
	**% (subgroup)**	83.3	16.7	100.0
	**% (sex)**	5.2	0.6	2.4
	**Total**	2.0	0.4	2.4
**Group D** **(Grade D RE)**	**Number**	2	7	9
	**% (subgroup)**	22.2	77.8	100.0
	**% (sex)**	1.0	2.3	1.8
	**Total**	0.4	1.4	1.8
**Total**	**Number**	192	311	503
	**% (subgroup)**	38.2	61.8	100.0
	**% (sex)**	100.0	100.0	100.0
	**Total**	38.2	61.8	100.0

**Table 3:** Comparison of the males and females regarding thickness and elasto values.

-	-	**Number**	**Mean**	**Standard Deviation**	**Minimum**	**Maximum**	***p***
**Age**	M	192	43.73	13.226	18	74	0.155
	F	311	41.88	14.648	18	78	
**Total wall thickness (mm)**	M	192	2.5760	0.55139	1.40	4.30	<0.001
	F	311	2.2717	0.47203	1.00	3.90	
**Muscularis propria thickness (mm)**	M	192	1.1203	0.30296	0.50	2.20	<0.001
	F	311	0.9929	0.24655	0.30	1.80	
**Submucosa-mucosa thickness (mm)**	M	192	1.4573	0.32351	0.80	2.40	<0.001
	F	311	1.2804	0.28210	0.70	2.30	
**Muscularis Propria SWV (m/s)**	M	192	2.0603	0.28316	1.24	2.99	<0.001
	F	311	2.2818	0.26515	1.59	3.30	
**Submucosa-mucosa SWV (m/s)**	M	192	1.8993	0.26919	1.15	2.92	<0.001
	F	311	2.1324	0.26023	1.50	2.95	
**Total wall SWV (m/s)**	M	192	1.9798	0.27108	1.19	2.96	<0.001
	F	311	2.2071	0.25639	1.57	3.00	

**Table 4:** The comparison of RE (+) and RE (-) patients regarding age, thickness, and elasto values.

-		**Number**	**Mean**	**Standard deviation**	**Minimum**	**Maximum**	***p***
**Age**	Group N	374	41.20	13.944	18	77	<0.001
	Group RE	129	46.61	19.974	18	78	
	Total	503	42.59	14.137	18	78	
**Total wall thickness (mm)**	Group N	374	2.3222	0.47671	1.30	4.00	<0.001
	Group RE	129	2.5783	0.60647	1.00	4.30	
	Total	503	2.3879	0.52457	1.00	4.30	
**Muscularis propria thickness (mm)**	Group N	374	1.0107	0.24852	0.40	1.90	<0.001
	Group RE	129	1.1310	0.32925	0.30	2.20	
	Total	503	1.0416	0.27622	0.30	2.20	
**Submucosa-mucosa thickness (mm)**	Group N	374	1.3078	0.28804	0.70	2.30	<0.001
	Group RE	129	1.4643	0.34319	0.80	2.40	
	Total	503	1.3479	0.31043	0.70	2.40	
**Muscularis Propria SWV (m/s)**	Group N	374	2.2888	0.26008	1.24	3.30	<0.001
	Group RE	129	1.9318	0.20679	1.40	2.36	
	Total	503	2.1973	0.29244	1.24	3.30	
**Submucosa-mucosa SWV (m/s)**	Group N	374	2.1304	0.26033	1.15	2.95	<0.001
	Group RE	129	1.7913	0.19718	1.25	2.34	
	Total	503	2.0435	0.28678	1.15	2.95	
**Total wall SWV (m/s)**	Group N	374	2.2096	0.25382	1.19	3.00	<0.001
	Group RE	129	1.8616	0.19559	1.37	2.35	
	Total	503	2.1204	0.28420	1.19	3.00	

**Table 5:** Inter-features correlation coefficients.

-	**Age**	**Total wall thickness**	**Muscularis propria thickness**	**Submucosa-mucosa thickness**	**Muscularis Propria SWV**	**Submucosa-mucosa SWV**	**Total wall SWV**
**Age**	1						
**Total wall thickness**	0.317**	1					
**Muscularis propria thickness**	0.213**	0.865**	1				
**Submucosa-mucosa thickness**	0.363**	0.880**	0.571**	1			
**Muscularis Propria SWV**	-0.101*	-0,151**	-0.134**	-0.150**	1		
**Submucosa-mucosa SWV**	-0.096*	-0.205**	-0.173**	-0.199**	0.926**	1	
**Total wall SWV**	-0.100*	-0.181**	-0.156**	-0.178**	0.982**	0.981**	1

**Note:** *p<0.05, **p<0.01

**Table 6:** Comparison of features between normal group and subgroups.

-	-	**Number**	**Mean**	**Standard Deviation**	**Min**	**Max**	*p*
**Age**	**Group N**	374	41.20 b	13.944	18	77	<0.001
	**Group A**	80	46.19 ab	13.377	18	78	
	**Group B**	28	45.25 ab	13.462	20	65	
	**Group C**	12	52.33 a	14.625	32	72	
	**Group D**	9	47.00 ab	20.112	20	72	
**Total wall thickness (mm)**	**Group N**	374	2.3222 bc	0.47671	1.30	4.00	<0.001
	**Group A**	80	2.6287 ab	0.58031	1.30	4.30	
	**Group B**	28	2.4571 abc	0.65287	1.00	3.70	
	**Group C**	12	2.7833 a	0.60578	1.70	3.80	
	**Group D**	9	2.2333 c	0.58523	1.60	3.10	
**Muscularis propria thickness (mm)**	**Group N**	374	1.0107 ab	0.24852	0.40	1.90	<0.001
	**Group A**	80	1.1550 a	0.33599	0,30	2.20	
	**Group B**	28	1.1000 ab	0.32660	0.50	1.80	
	**Group C**	12	1.1833 a	0.32146	0.60	1.70	
	**Group D**	9	0.9444 b	0.25550	0.60	1.40	
**Submucosa-mucosa thickness (mm)**	**Group N**	374	1.3078 b	0.28804	0.70	2.30	<0.001
	**Group A**	80	1.4788 ab	0.33440	0.80	2.30	
	**Group B**	28	1.4214 ab	0.33038	0.80	2.10	
	**Group C**	12	1.6000 a	0.39543	1.00	2.40	
	**Group D**	9	1.2889 b	0.35512	0.90	1.80	
**Muscularis propria SWV (m/s)**	**Group N**	374	2.2888 a	0.26008	1.24	3.30	<0.001
	**Group A**	80	1.9581 b	0.20293	1.46	2.36	
	**Group B**	28	1.8721 b	0.21521	1.40	2.29	
	**Group C**	12	1.8725 b	0.19132	1.55	2.21	
	**Group D**	9	1.9622 b	0.21247	1.63	2.36	
**Submucosa-mucosa SWV (m/s)**	**Group N**	374	2.1304 a	0.26033	1.15	2.95	<0.001
	**Group A**	80	1.8045 b	0.19500	1.25	2.34	
	**Group B**	28	1.7707 b	0.20487	1.40	2.17	
	**Group C**	12	1.7125 b	0.16896	1.44	2.01	
	**Group D**	9	1.8433 b	0.22382	1.50	2.18	
**Total wall SWV (m/s)**	**Group N**	374	2.2096 a	0.25382	1.19	3.00	<0.001
	**Group A**	80	1.8813 b	0.19086	1.37	2.35	
	**Group B**	28	1.8214 b	0.20768	1.40	2.23	
	**Group C**	12	1.7925 b	0.17499	1.50	2.11	
	**Group D**	9	1.9028 b	0.21470	1.57	2.27	

## Data Availability

All the data and supportive information is provided within the article.

## LIST OF ABBREVIATIONS

RE: Reflux Esophagitis
SWV: Shear Wave Velocity
TWT: Total Wall Thickness
GERD: Gastroesophageal reflux disease
CT: Computed Tomography
MRI: Magnetic Resonance Imagıng

## STANDARDS OF REPORTING

STROBE guidelines were followed.
